# Supplementary material for: Novel NFκB Inhibitor SC75741 Mitigates Chondrocyte Degradation and Prevents Activated Fibroblast Transformation by Modulating miR-21/GDF-5/SOX5 Signaling
Source: Int J Mol Sci. 2021 Oct 14;22(20):11082. doi: 10.3390/ijms222011082 (PMC8538686; doi:10.3390/ijms222011082)
Supplement: Supplementary file 1 [file ijms-22-11082-s001.zip › ijms-1397959-supplementary.pdf]

SUPPLEMENTARY INFORMATION

Novel NFκB Inhibitor SC75741 Mitigates Chondrocyte Degra-dation and Prevents Activated Fibroblast Transformation by Modulating miR-21/GDF-5/SOX5 Signaling

Pei-Wei Weng [1,2,3], Vijesh Kumar Yadav [4], Narpati Wesa Pikatan [4], Iat-Hang Fong [4], I-Hsin Lin [5,6*], Chi-Tai Yeh [4,7] and Wei-Hwa Lee [4,8*]

[1] Department of Orthopaedics, School of Medicine, College of Medicine, Taipei Medical University, Taipei City 110, Taiwan.

[2] Department of Orthopaedics, Shuang Ho Hospital, Taipei Medical University, New Taipei City 23561, Taiwan.

[3] Graduate Institute of Biomedical Materials and Tissue Engineering, College of Biomedical Engineering, Taipei Medical University, Taipei City 110, Taiwan.

[4] Department of Medical Research & Education, Taipei Medical University - Shuang Ho Hospital, New Taipei City 23561, Taiwan.

[5] School of Dentistry, College of Oral Medicine, Taipei Medical University, Taipei City 110, Taiwan.

[6] Division of Periodontics, Department of Dentistry, Taipei Medical University - Shuang Ho Hospital, New Taipei City 23561, Taiwan.

[7] Department of Medical Laboratory Science and Biotechnology, Yuanpei University of Medical Technology, Hsinchu City 30015, Taiwan.

[8] Department of Pathology, Taipei Medical University-Shuang Ho Hospital, New Taipei City 23561, Taiwan.

* Correspondence: author(s): I-Hsin Lin, MD, Division of Periodontics, Department of Dentistry, Taipei Medical University - Shuang Ho Hospital, New Taipei City 23561, Taiwan. Tel: +886-2-2490088 ext. 8881, Fax: +886-2-2248-0900. E-mail: 14198@s.tmu.edu.tw ; Wei-Hwa Lee, MD., Ph.D, Department of Pathology, Taipei Medical University-Shuang Ho Hospital, New Taipei City, Taiwan. Tel: +886-2-2490088 ext. 8885, Fax:886-3-5401480. E-mail: whlpath97616@s.tmu.edu.tw

Supplementary Table S1. Primer sequence in this study.

Gene		sequence
IL-6	forward	AGACAGCCACTCACCTCTTCAG
	reverse	TTCTGCCAGTGCCTCTTTGCTG
COX2	forward	CGGTGAAACTCTGGCTAGACAG
	reverse	GCAAACCGTAGATGCTCAGGGA
NFκB	forward	GCAGCACTACTTCTTGACCACC
	reverse	TCTGCTCCTGAGCATTGACGTC
TNF-a	forward	CTCTTCTGCCTGCTGCACTTTG
	reverse	ATGGGCTACAGGCTTGTCACTC
miR-21	forward	ACGTTGTGTAGCTTATCAGACTG
	reverse	AATGGTTGTTCTCCACACTCTC

Supplementary Table S2. The membranes were incubated in primary antibodies

No.	Target	Dilution	Source		
1	TNF-α	1:1000	25	#8184	Cell Signaling
2	NFκB	1:1000	65	#8242	Cell Signaling
3	IL-6	1:1000	21-28	#12153	Cell Signaling
4	Cox-2	1:1000	74	#12282	Cell Signaling
5	COL4A1	1:1000	200	#50273	Cell Signaling
6	SOX5	1:1000	84	ab94396	Abcam
7	GDF-5	1:1000	13	ab166617	Abcam
8	TGF- β1	1:1000	44	ab215715	Abcam
9	MMP9	1:1000	78	ab76003	Abcam
10	RANKL	1:1000	35	ab9957	Abcam
11	TRAP	1:1000	37	ab52750	Abcam
12	OPG	1:1000	46	ab73400	Abcam
13	α-SMA	1:1000	42	ab7817	Abcam
14	ACAN	1:1000	200	sc-33695	santa cruz
15	β-actin	1:10000	42	66009-1-Ig	proteintect

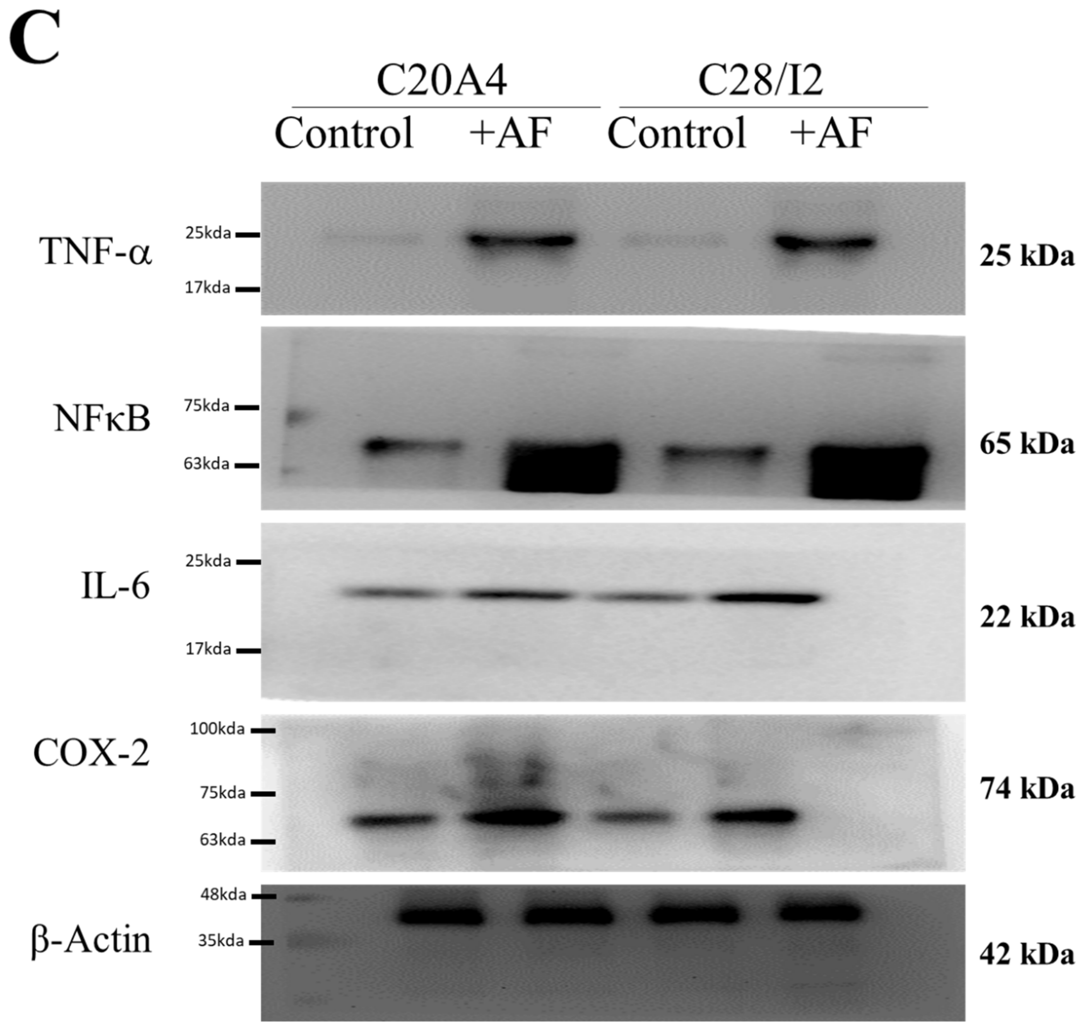

Supplementary Figure S1. Full-size blots of Figure 1C

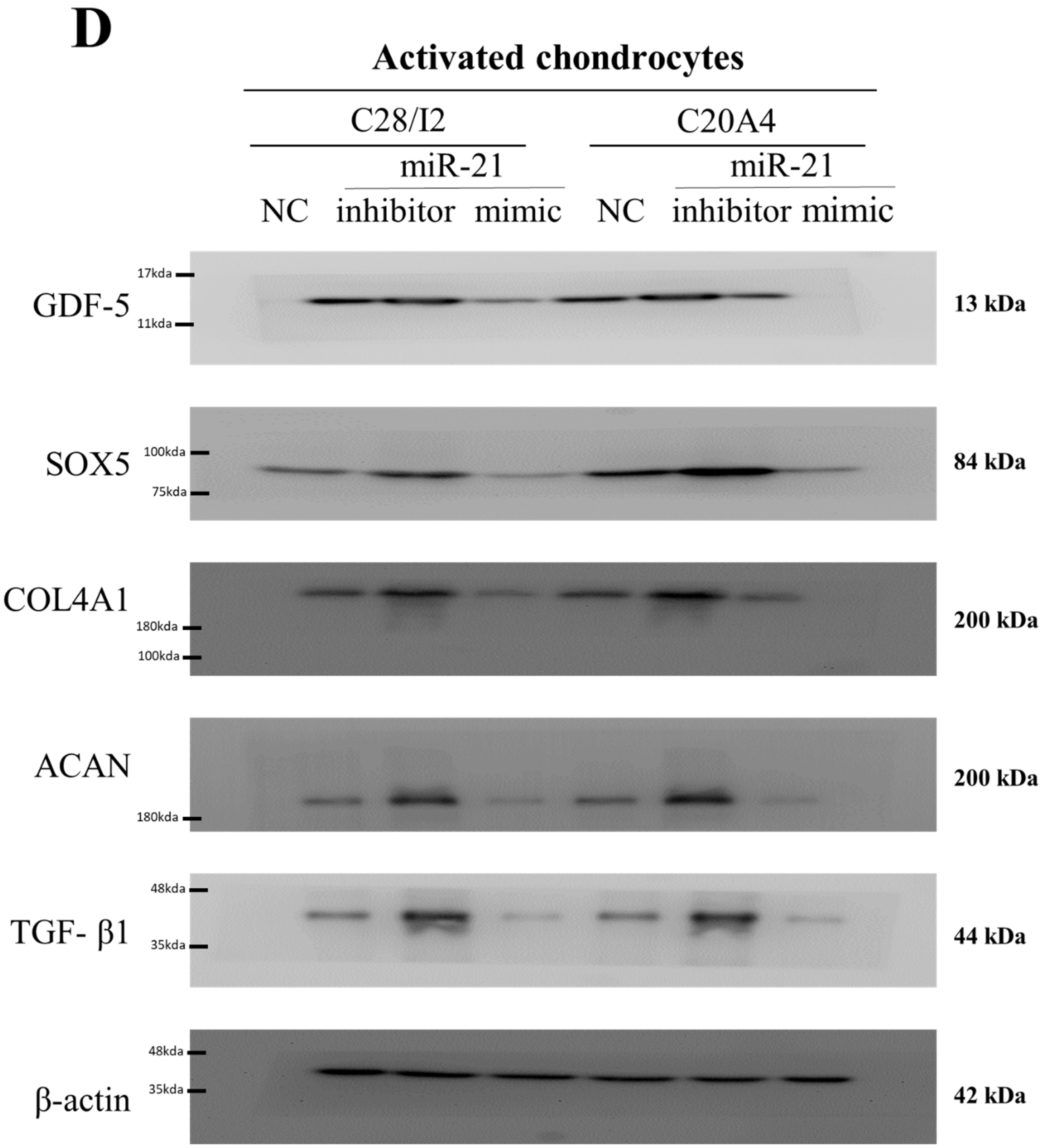

Supplementary Figure S2. Full-size blots of Figure 2D

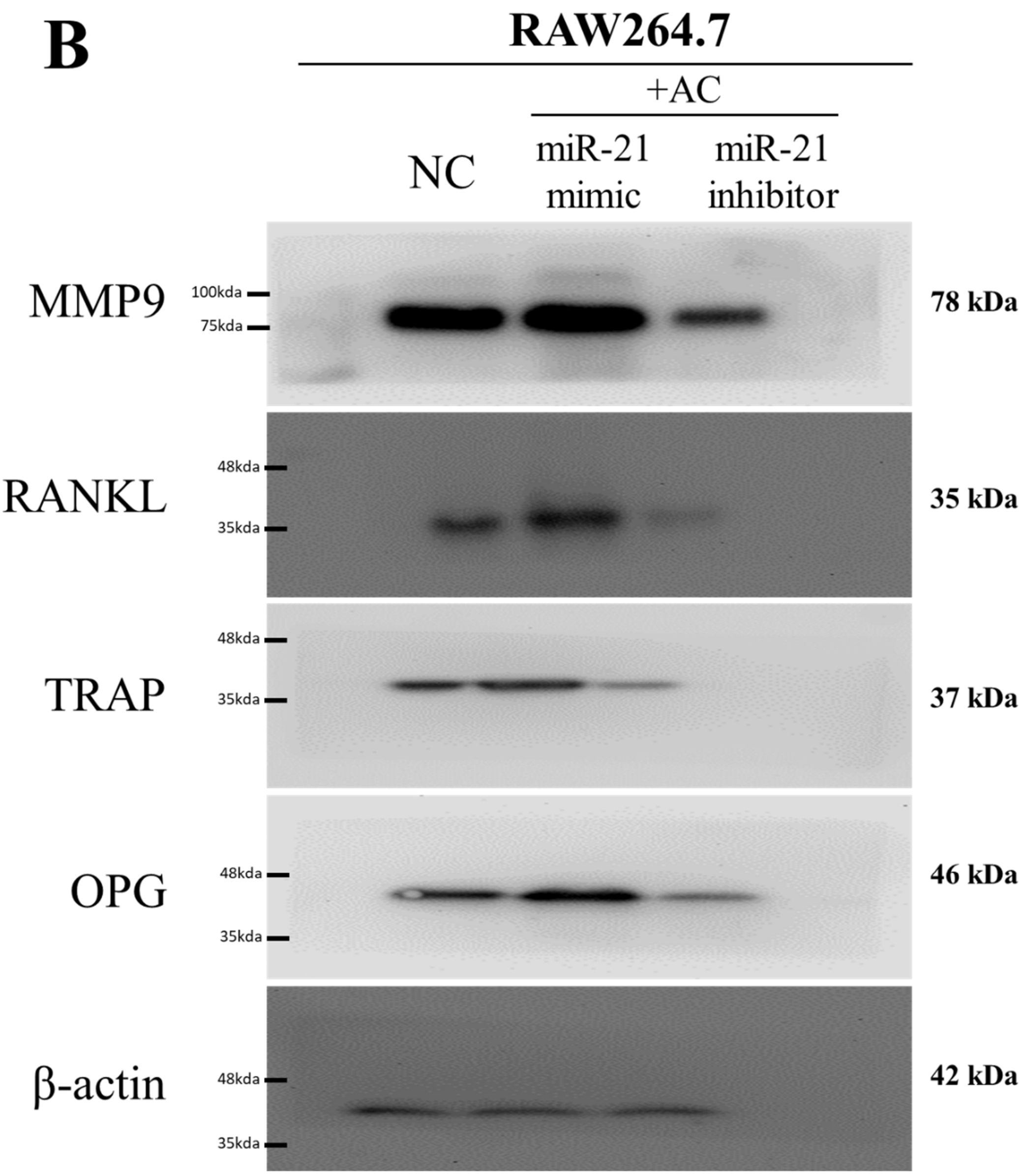

Supplementary Figure S3. Full-size blots of Figure 3B

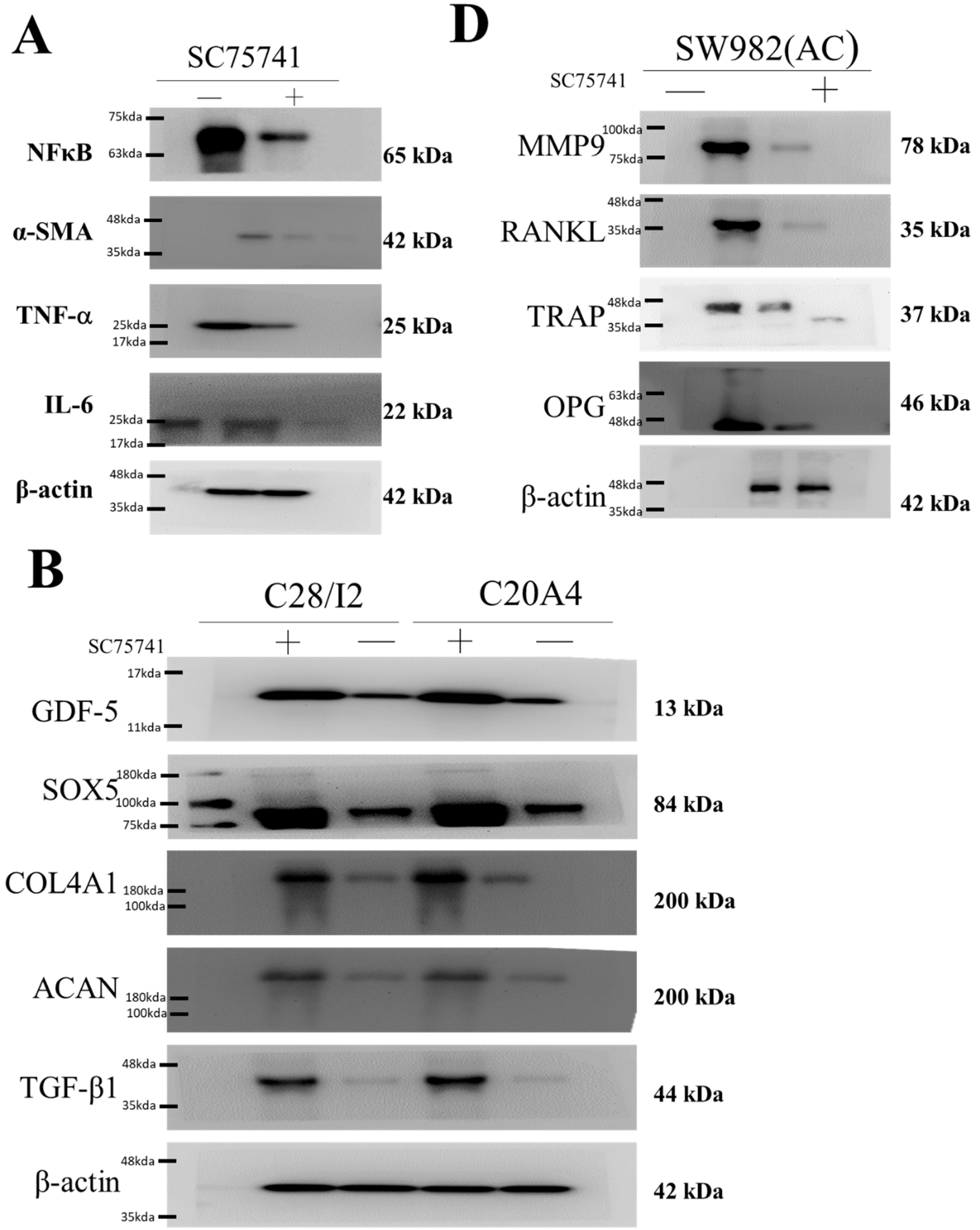

Supplementary Figure S4. Full-size blots of Figure 4A, 4B and 4D
